# The Adaptive Behavioral Components (ABC) Model for Planning Longitudinal Behavioral Technology-Based Health Interventions: A Theoretical Framework

**DOI:** 10.2196/15563

**Published:** 2020-06-26

**Authors:** Sean D Young

**Affiliations:** 1 Institute for Prediction Technology Department of Informatics University of California, Irvine Irvine, CA United States; 2 Department of Emergency Medicine UCI School of Medicine Irvine, CA United States

**Keywords:** health behavior, risk behavior, behavioral medicine, public health informatics, consumer health informatics, psychological theory

## Abstract

A growing number of interventions incorporate digital and social technologies (eg, social media, mobile phone apps, and wearable devices) into their design for behavior change. However, because of a number of factors, including changing trends in the use of technology over time, results on the efficacy of these interventions have been mixed. An updated framework is needed to help researchers better plan behavioral technology interventions by anticipating the needed resources and potential changes in trends that may affect interventions over time. Focusing on the domain of health interventions as a use case, we present the Adaptive Behavioral Components (ABC) model for technology-based behavioral interventions. ABC is composed of five components: basic behavior change; intervention, or problem-focused characteristics; population, social, and behavioral characteristics; individual-level and personality characteristics; and technology characteristics. ABC was designed with the goals of (1) guiding high-level development for digital technology–based interventions; (2) helping interventionists consider, plan for, and adapt to potential barriers that may arise during longitudinal interventions; and (3) providing a framework to potentially help increase the consistency of findings among digital technology intervention studies. We describe the planning of an HIV prevention intervention as a case study for how to implement ABC into intervention design. Using the ABC model to plan future interventions might help to improve the design of and adherence to longitudinal behavior change intervention protocols; allow these interventions to adapt, anticipate, and prepare for changes that may arise over time; and help to potentially improve intervention behavior change outcomes. Additional research is needed on the influence of each of ABC’s components to help improve intervention design and implementation.

## Introduction

Digital and social technologies (eg, social media, smartphone apps, and wearable devices) have promising potential for achieving rapid and widespread health behavior change. Social media has been used to change and predict a number of health-related behaviors, including HIV testing and sexually transmitted diseases, suicide prevention, car crashes, and opioid-related emergency department visits [1-7]; Fitbits and self-tracking devices have been proposed as intervention tools to increase exercise [8,9] and reduce stress; health systems and insurers have integrated wearable device, social media and patient health/medical data to try to improve clinical outcomes [10,11]; and smartphone apps have be studied for their potential to improve a variety of health behaviors and outcomes, such as weight loss and diabetes self-management [12-14].

However, results have been mixed on whether and how digital technologies might change people’s health behaviors [15-20]. Although some of these differences may be related to common methodological and study assessment–related reasons (eg, differences in study duration or outcomes), there are also a number of potential intervention design–related reasons for these inconsistences. For example, a large volume of behavioral psychology research suggests that small contextual changes have a dramatic effect on behavior [21-24], such as the size of a button on a website, or the way in which information is communicated and displayed. Although contextual issues can and have been found to impact intervention success regardless of whether it is an online or offline study (eg, age, race/ethnicity, and sex of experimenter may affect participant adherence and engagement), these issues become more complicated when delivering interventions digitally because of the frequency and ease of changing variables in digital technologies compared with offline interventions (eg, placement of buttons and text, inclusion of social interaction, and gamification), as well as reliance on technology companies (eg, Facebook and Twitter) to maintain similar and stable versions of their products. These issues have become increasingly important with the current COVID-19 pandemic, as there is growing need to integrate new technologies into research and clinical care to address COVID-19 policies, such as social distancing and stay-at-home orders.

Due to changing trends in design and use of digital technologies, it is, therefore, extremely difficult to exactly replicate a technology-based intervention study, as the earlier study might have occurred on a now outdated software platform. This creates an additional problem as grant applications often require planning 3- to 5-year studies in advance despite the potential technological changes that may occur during that period. How can researchers designing digital technology–based interventions address, or at least anticipate, these issues? New theories are needed to build on existing health informatics and behavioral technology-based intervention models [25].

This manuscript proposes a theoretical framework, called the Adaptive Behavioral Components (ABC) theory for technology-based interventions, with the goals of (1) guiding high-level development for digital health technology–based interventions; (2) helping interventionists consider, plan for, and adapt to potential barriers that may arise during longitudinal interventions; and (3) providing a framework to potentially help increase consistency of findings among digital technology intervention studies. We seek to describe and synthesize categories of prior research (below) into a new theory to help researchers design digital health technology–based interventions.

**Figure 1:**
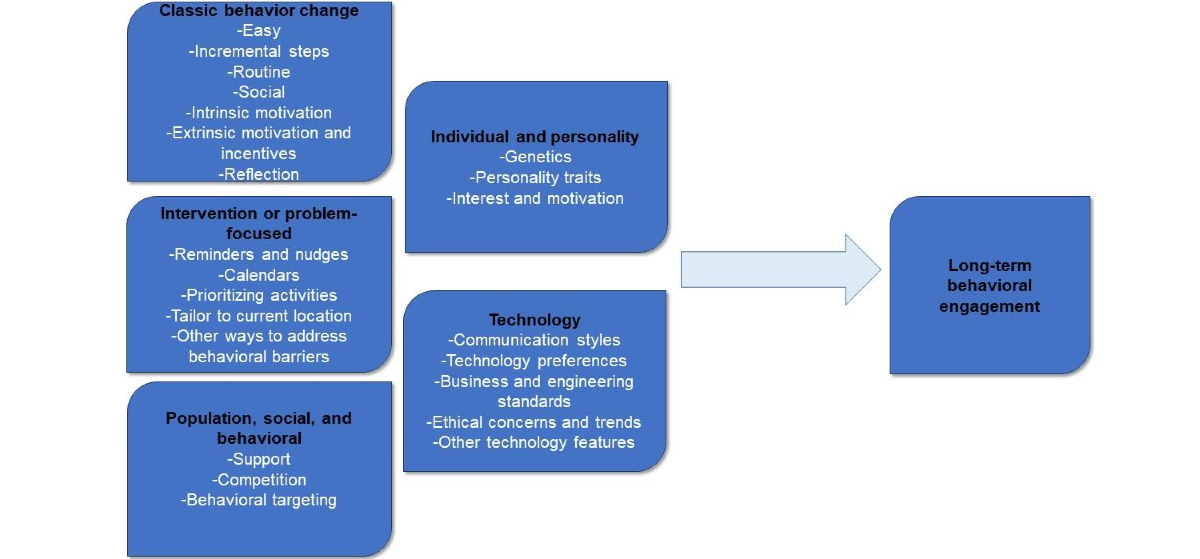
Adaptive Behavioral Components Theory (ABC) for Technology-based Interventions. By helping researchers to consider these five high-level components, the model provides guidance on how to plan for the needs and potential changes during a technology-based health intervention.

ABC incorporates research across the fields of social and behavioral psychology, informatics, and marketing to develop a model tailored to the needs of digital technology interventionists. The model is based on five overarching factors needed for guiding a sustainable technology-based intervention: (1) basic behavior change components; (2) intervention and problem-focused characteristics; (3) population, social, and behavioral characteristics; (4) individual-level and personality characteristics; and (5) technology characteristics (Figure 1).

A large body of research, including theoretical modeling, has been conducted on many of these five areas. ABC is not meant to replace existing theories specific to one or more of these components, but rather to provide a guide for when and how to think about the inter-related, diverse, and overarching concepts affecting behavior change that are often not considered within behavioral interventions. The first four components of ABC can apply broadly to behavioral interventions, whereas the last component is specific to digital technology–based interventions. Although the model might also be applied outside of health, this manuscript focuses on health applications to narrow the scope of discussion and provide context. The five components of ABC are described in more detail below, along with a case study of how it has been learned and might be applied in research settings.

## The ABC Framework

### Basic Behavior Change Components

There are a number of behavior change methods and theories, such as social normative and social cognitive theory–based interventions, that have been broadly and successfully applied to change health behaviors, including HIV risk behaviors [26] and smoking cessation [27]. These methods are typically rooted in social and behavioral psychological theory [20] and broadly suggest that interventions will be more efficacious if they (1) make it easy to engage in the target behavior [28-32], (2) make the behavior routine rather than infrequent and unpredictable [33,34], (3) incorporate social components such as support and/or competition [2,35-37], (4) build change incrementally (eg, dieters who focused on the daily steps of eating healthy were more likely to adhere to their healthy eating routine compared with those who focused on the end goal of losing weight [and these individuals actually gained more weight at follow-up]) [38], (5) develop intrinsic [39-41] and (6) extrinsic motivation (being cautious about the sometimes negative effects of incentives on behavior) [42,43], and (7) provide ways for people to reflect on their progress [44,45]. Integration of these basic behavior change components may help to improve intervention behavior change–related outcomes.

### Intervention/Problem-Focused Characteristics

Interventions may benefit from further tailoring to address the *specific* behavioral barriers preventing people from conducting the intended behavior. These intervention or problem-focused characteristics are typically contextual factors that affect people’s willingness and ability to participate and remain engaged in an intervention. For example, people might not engage in behaviors because of a variety of reasons, including lack of remembering, interest, or time; belief that the intervention is unlikely to help or may harm them; cost; or that the intervention is ill-suited to their lifestyle, values, or expectations. The specific intervention framework should be guided by these needs [46]. For example, an intervention designed to send mobile-delivered medication reminders would likely be more beneficial to individuals who are forgetting to take their medication compared with individuals who fear the side effects of the medication. Similarly, environmental and immediate contextual-level tailoring (eg, just-in-time interventions) may benefit individuals who need to be reminded to engage in activities at certain times or in certain locations [47]. For example, a text message reminder can be sent as a reminder tool to attempt to prevent drug use for a substance-using individual who is walking in an area detected by the phone to be a high-risk area for substance use, an example of timecasting [48]. By understanding the reasons why people are not engaging in a specific behavior, intervention characteristics can be tailored to optimize addressing that barrier to behavior change.

### Population/Social/Behavioral Characteristics

Tailoring interventions to people’s differences in population, social, and behavioral characteristics may help to further improve intervention efficacy. For example, tailoring intervention content to the target population (eg, creating racially and culturally tailored online recruitment advertisements for a study among African Americans) may increase recruitment efficiency [49]. In a study demonstrating this approach, African American men had increased odds of clicking on a Web-based advertisement that included an image of an African American model vs a white model [50]. Similarly, tailoring interventions to include individuals who share similar demographic characteristics and preferences (ie, peers) may be more efficacious compared with interventions that do not tailor for these factors [51]. Tailoring interventions to an individual’s behavioral state might also lead to higher engagement compared with not including this tailoring. For example, online smoking cessation health information may have higher click-through and engagement rates if delivered to people who have been searching on Google for ways to quit smoking compared with individuals browsing on Facebook to talk with friends.

### Individual-Level/Personality Characteristics

Despite the success of social- and community-based interventions, many people within these interventions do not follow the group norm because of individual-level differences, such as differences in experience, genetics, personality, and disposition [52]. For example, in a study on factors affecting health, the personality factor, that is, conscientiousness, predicted all study outcomes, with individuals identified as being less responsible and less self-controlled tending to report poorer health, be more overweight, and engage in more substance use. Tailoring interventions at these individual levels, such as by targeting familial, genetic, and/or personality characteristics, might increase intervention efficacy. For example, participants who have personality traits identifying them as “more social” might be targeted as peer role models for peer-led interventions [53-55]. Similarly, individuals with genetic predisposition for alcohol use disorder [56] might be targeted for an alcohol-related intervention to help researchers learn more about genetic factors/predisposition and improve targeting of at-risk individuals. However, research on the role and methods of developing interventions based on individual-level/personality characteristics has been limited, leading researchers to call for additional research on this topic [57].

### Technology Characteristics

Although the above four factors affect and should be considered when conducting any type of behavior change intervention, the technology characteristics component is specific and critical to the success of *digital technology* interventions. It is also perhaps the component that requires the most planning of all the five factors, because of the changing needs and trends in technology use. Intervention engagement can be affected by a variety of technology characteristics, such as current trends in popular communication style (eg, use of video as a preferred communication medium vs text), changes in features of a technology (eg, adding an online community feature to a software app may change user engagement rates), and changes in ethical concerns (eg, social media engagement may decline after a recent security breach on that social media platform) [58]. These factors might have a large impact on intervention efficacy. Understanding and preparing for technology characteristics and potential adaptations is, therefore, essential for the delivery of effective longitudinal technology-based interventions.

## Discussion

### Lessons Learned and How to Apply

As a case study identifying the five components of ABC and how to apply them, we will describe the process for planning an intervention to increase the initiation of pre-exposure prophylaxsis (PrEP) as a method of HIV prevention among African American and Latino men who have sex with men (MSM) who are aged 18 years or older and living in Los Angeles. The primary behavior we wish to change is, therefore, to increase the number of participants initiating PrEP. Table 1 can be used as a reference for when and how the ABC framework can be incorporated into planning this intervention.

**Table 1:** Applying the Adaptive Behavioral Components (ABC) model for technology-based interventions. This table provides an example of how a researcher might apply the ABC framework in an intervention designed to increase uptake of pre-exposure prophylaxis among African American and Latino men who have sex with men at risk for HIV. The examples below are not exhaustive but are meant to assist readers in understanding how to integrate ABC into intervention design.

Category and subcategory		Example
**Classic behavior change**		
	Easy	Provide online diagnosis and prescription referral as an option to increase PrEP^a^ accessibility
	Incremental steps	Gradually discuss and promote PrEP with participants, beginning with friendly topics first to gain trust
	Routine	Routine (eg, weekly) communications with study participants
	Social	Encourage discussions and social interaction among participants
	Intrinsic motivation	Educate participants on the importance of prevention and safe sex
	Extrinsic motivation	Provide products/services that have tangible value to participants (eg, free PrEP and HIV self-test)
	Reflection	Encourage participants to talk about their experiences using PrEP so that they reflect on and think about it
**Intervention/problem-focused**		
	Reminders/nudges	Remind participants to make an appointment and use PrEP
	Calendars	Codevelop a calendar with them with dates for when they will schedule an appointment
	Prioritizing activities	Cocreate a list of priorities with them to help them fit a PrEP medical visit and use into their priorities
	Tailor to current location	Discuss PrEP when participants are available based on their location and schedule
**Population/social/behavioral**		
	Social support	Develop a peer support network where peer role models deliver the intervention
	Competition	Integrate friendly games
	Behavioral targeting	Provide recruitment advertisements and/or HIV prevention information to participants at the right time and context (eg, when participants are requesting or searching for information about safe sex)
**Individual/personality**		
	Genetics	Tailor the intervention based on genetic differences in response found in research
	Personality traits	Tailor the intervention based on personality differences in response found in research
	Interest/motivation	Tailor the intervention based on interest/motivation (eg, individuals who are less interested may need greater financial incentives)
**Technology**		
	Communication styles	Offer multiple methods of communication (eg, chat, video, and text message)
	Technology preferences	Deliver the intervention on platforms that are already being used by minority men who have sex with men (ie, the target population)
	Business/engineering standards	Awareness and planning based on current and potential future changing trends in the technology platforms being used
	Ethical considerations/trends	Awareness and planning technology features that address current and potential future ethical considerations (eg, for an HIV prevention intervention, private communication is important to reduce experience of stigma)
	Other technology features	Deliver the intervention on multiple technology platforms to avoid risks from one platform

^a^PrEP: pre-exposure prophylaxsis.

Although there are numerous reasons why individuals do not take PrEP, using the ABC framework to analyze the intervention/problem-focused characteristics teaches us that we need to narrow to focus on only one of these reasons (stigma) and develop an intervention designed to decrease stigma to increase PrEP use. As stigma is a social construct (created and perpetuated by one’s peers) [59], we seek to counteract stigma that prevents people from using PrEP by creating an intervention that will leverage peers to promote PrEP uptake (ie, we will deliver a peer-led, community-based intervention). Within the intervention framework, we will attempt to include many of the basic behavior change components. For example, we will make it easy for participants to request PrEP by allowing them to receive an online tele-consultation and request PrEP online, making it easier (and less stigmatizing) than typical methods of having to visit and get tested at in-person clinics. We will also hire peer leaders to gradually become friends and then promote PrEP behavior change with participants each week, incentivizing routine discussions with the peer leaders.

We will integrate the population/social/behavioral characteristics of ABC by designing recruitment materials, intervention content, and processes that are tailored for this population (eg, recruitment advertisements include sexually provocative images of minority MSM; advertisements will be placed on websites where minority MSM are already searching for safe sex information [eg, Google ad words]; and HIV prevention conversations will be delivered by minority MSM peer leaders and focused on issues relevant to minority MSM).

Although the intervention is designed for African American and Latino MSM, we anticipate individual differences such that not all individuals will respond to the intervention as planned. To address this, we will meet peer leaders each week to learn about the barriers and facilitators in promoting PrEP uptake among each individual participant, to refine and further tailor the intervention to individuals who are not responsive to the intervention (eg, some participants may require more personal time/education than the average group member).

Finally, we wish to use technologies to scale the peer-led PrEP uptake intervention because of a technology-based intervention’s potential for rapidly and cost-effectively scaling behavior change. We seek to use social media and mobile social technologies to deliver the intervention because of their alignment with and ability to meet the requirements stated above (eg, social media technologies allow African American and Latino MSM peer community leaders to communicate with and spread PrEP uptake–related behavior change information to other African American and Latino MSM both within a [private] group and/or individually). The Harnessing Online Peer Education (HOPE) intervention, a peer-led online community-based behavior change intervention, is one example of this type of intervention and one that we seek to use. Although HOPE interventions have been focused on different populations and behavioral outcomes, including HIV testing (HOPE HIV), mental health, and opioid misuse, with efficacy demonstrated in changing primary behavioral outcomes [2,51,60,61], all members of this family of intervention rely on using social technologies as a method for delivering a social normative (eg, peer-based) intervention.

However, deciding the specific technology component(s) to be used in HOPE is perhaps the most important and difficult aspect in our study plan because of the changing trends in technologies.

For example, a multiwave, 5-year HOPE HIV testing intervention was funded in 2014 based on earlier methods and results from HOPE interventions conducted in 2010 and 2012 that showed success in using Facebook groups as an intervention delivery platform [2,51]. However, from 2010 to 2014, and throughout the 5 years following 2014, a large number of changes have occurred on social media, including Facebook no longer being the sole dominant technology used for online communities or forums among all age groups and populations. With the introduction of other platforms, such as Snapchat and Instagram, use of Facebook has waned, especially among youth [62], which might reduce the engagement and potential efficacy of interventions delivered on Facebook that rely on previous rates of engagement. Similarly, throughout a 5-year period, Facebook has made a number of changes to their interface, affecting HOPE intervention engagement.

For example, in 2018, Facebook’s Mark Zuckerberg made a public statement that Facebook would be changing the way their algorithm displayed content, upsetting many individuals and businesses who had relied on previous methods for posting on Facebook that might no longer work [63]. Previous longitudinal behavioral interventions on Facebook were impacted by these changes. For example, in an HIV prevention intervention run by our team using Facebook groups during this period, we have been finding that Facebook platform changes have resulted in intervention participants receiving fewer notifications about HIV prevention and testing compared with control (Facebook) group participants. Although the intervention group remains significantly more engaged in posting compared with the control group (as intended by the intervention), the large number of posts within the intervention group combined with the changes to the way Facebook groups and the Facebook algorithm are used initially resulted in fewer testing-related posts being viewed by the intervention group compared with the control group. As a result, our team has learned the importance of delivering technology-based interventions through multiple technology platforms to minimize the impact on the intervention because of changes that may occur on one of the technology platforms or by changing trends in the types of technologies used by participants.

We responded to these experiences by learning that we need to modify the way technology-based interventions are designed and delivered. In the future, we seek to develop a HOPE intervention that leverages multiple platforms, including Facebook, Instagram, Snapchat, and YouTube (or whichever platforms that are dominant at that time), and ensures that the elements of ABC can be integrated into one or more of these platforms. By delivering the intervention across multiple platforms, we seek to (1) reduce the risk that changes in one or more of the technology platforms alters intervention delivery; (2) reduce the risk that major changes to the study protocol might be needed at a later period; (3) allow flexibility within the study protocol for it to be tailored to incorporate additional social technologies in the future (eg, by leveraging multiple technologies to deliver the intervention, each of these play a smaller role and allows for technologies to be replaced over time as needed with trends while keeping the scope of work intact); (4) allow the ability to simultaneously tailor the intervention to different subdemographic groups and populations (ie, tailoring for age, race, and ethnicity based on the social technologies used most frequently by those groups); and (5) allow the ability to incorporate multimedia methods and trends in communication, such as use of social technologies that are designed for sharing of text, images, videos, and/or other methods such as virtual reality.

We also recommend to our own team and other researchers to track every available relevant digital metric (eg, click-through rates, downloads, and likes) and to conduct a pilot study before proceeding with an intervention. These digital data can be very useful in informing potential unknown problems that might occur during an intervention [64]. They are also important as they can help monitor and address unforeseen circumstances that may occur in the middle of a digital intervention (eg, a prolonged drop in engagement on a digital platform may be a signal for the need to shift the intervention methods/content to a different digital platform). Taken together, we have learned the importance of both accounting for changes in technology use and the need to anticipate and adapt various behavior change components across technologies to improve the efficacy and durability of digital behavioral interventions.

### Conclusions

The ABC model was designed as a guide both to assist in planning digitally delivered behavior change interventions and to help anticipate the needed resources and potential changes in trends that may affect an intervention over time. Using the ABC model to plan future interventions may help to improve the design of and adherence to longitudinal behavior change intervention protocols; allow these interventions to adapt, anticipate, and prepare for changes that may arise over time; and help to potentially improve intervention behavior change outcomes.

## Abbreviations

ABC: Adaptive Behavioral Components
HOPE: Harnessing Online Peer Education
MSM: men who have sex with men
PrEP: pre-exposure prophylaxis
